# Surgical methods of treatment for cholecystolithiasis combined with choledocholithiasis: six years’ experience of a single institution

**DOI:** 10.1007/s00464-021-08843-x

**Published:** 2021-11-03

**Authors:** Tong Guo, Lu Wang, Peng Xie, Zhiwei Zhang, Xiaorui Huang, Yahong Yu

**Affiliations:** Department of Biliary-Pancreatic Surgery, Affiliated Tongji Hospital, Tongji Medical College, Huazhong University of Science and Technology, 1095 Jiefang Avenue, Wuhan, 430030 Hubei China

**Keywords:** Cholecystolithiasis, Choledocholithiasis, Surgical methods, Outcomes

## Abstract

**Introduction:**

The optimal treatment of choledocholithiasis combined with cholecystolithiasis remains controversial. Common surgical methods vary among endoscopic retrograde cholangiopancreatography (ERCP) followed by laparoscopic cholecystectomy (LC), laparoscopic transcystic common bile duct exploration (LTCBDE), laparoscopic transductal common bile duct exploration (LCBDE) with or without T-tube drainage. The purpose of this study is to evaluate the safety and effectiveness of surgical methods and to determine the appropriate procedure for patients with cholecystolithiasis combined with choledocholithiasis.

**Methods:**

From January 2013 to January 2019, a total of 1555 consecutive patients diagnosed with cholecystolithiasis combined with choledocholithiasis who underwent surgical treatment in Tongji Hospital were retrospectively analyzed. Total 521 patients with intrahepatic bile duct stones underwent LC + LCBDE + T-Tube were excluded from the analysis. At last, 1034 patients who met the inclusion criteria were divided into three groups according to their surgical methods: preoperative ERCP + subsequent LC (ERCP + LC group, *n* = 275), LC + LCBDE + intraoperative endoscopic nasobiliary drainage (ENBD) + primary duct closure (Tri-scope group, *n* = 479) and LC + laparoscopic transcystic CBD exploration (LTCBDE group, *n* = 280). Clinical records, operative findings and postoperative follow-up were collected and analyzed.

**Results:**

There was no mortality in three groups. Common bile duct (CBD) stone clearance rate was 97.5% in ERCP + LC group, 98.7% in Tri-scope group, and 99.3% in LTCBDE group. There were no difference in terms of demographic characteristics, biochemistry findings and presentations, but the Tri-scope group had the biggest diameter and amount of stones and diameter of CBD, the LTCBDE group had the least CBD stones and the biggest diameter of cystic gall duct (CGD). ERCP + LC group have the longest hospital stay (14.16 ± 3.88 days vs 6.92 ± 1.71 days vs 10.74 ± 5.30 days, *P* < 0.05), also has the longest operative time than others (126.08 ± 42.79 min vs 92.31 ± 10.26 min, 99.09 ± 8.46 min, *P* < 0.05). Compared to ERCP + LC group, LTCBDE group and Tri-scope group had lower postoperation-leukocyte, shorter surgery duration and hospital stay (*P* < 0.05). Compared to the Tri-scope group, the LTCBDE group had the shorter hospital stay, extubation time and operation time and less intraoperative bleeding. There were less postoperative complications in LTCBDE group (1.1%) compared to the ERCP + LC group (3.6%) and Tri-scope group (2.2%). Follow-up time was 6 to 72 months. Four patients in ERCP + LC group and 5 in Tri-scope group reported recurrent stones.

**Conclusion:**

All the three surgical methods are safe and effective. Tri-scope approach and LTCBDE approach have superiority to preoperative ERCP + LC. LC + LTCBDE shows priority over Tri-scope approach, but should be performed in selected patients. LC + LCBDE + T-Tube can be an alternative management if the other three procedures were failed. The surgeons should choose the most appropriate surgical procedure according to the preoperative examination results and intraoperative situation.

Common bile duct stones may occur in 10–18% patients undergoing laparoscopic cholecystectomy for cholecystolithiasis [1]. Choledocholithiasis can causes serious complications including pancreatitis, cholangitis and hepatic dysfunction on account of the biliary obstruction caused by stones [2]. Current treatment options for choledocholithiasis accompanied with cholecystolithiasis include preoperative endoscopic retrograde cholangiopancreatography (ERCP) and laparoscopic common bile duct exploration (LCBDE) via transcystic or open operation [3–5]. However, the optimal treatment for gallstones combined with common bile duct stones remains controversial. ERCP followed by LC became a widely accepted method but associated with postoperative complications in 5−11% of patients, including pancreatitis, hemorrhage, cholangitis, duodenal perforation and damage to function of the sphincter of Oddi [6]. With the advent of laparoscopy, LCBDE as a minimally invasive alternative to ERCP have the advantage of shorter hospital stays and lower cost [7–10]. LCBDE can be performed via transcystic approach or choledochotomy associated with T-tube drainage or primary duct closure. T-tube drainage of CBD has been generally adopted on the basis of experience in open surgery. Nevertheless, numerous published articles had reported T-tube drainage is associated with T-tube related complications such as bile leakage and electrolyte disturbances and inconvenience due to the indwelling of the T-tube. LTCBDE is appreciated by surgeons due to its lower biliary complications. However, the success rate of LTCBDE varies between 55 and 85% on account of the anatomy of cystic duct and size of CBD stones. In 2016, Estelle´s et al. conducted a retrospective study on LCBDE with primary closure after choledochotomy in 160 patients, of whom 11 patients (6.8%) had bile leakage [7]. In 2017, Pablo et al. conducted a comparative study of three bile duct closure methords (T-tube insertion, antegrade stenting, and primary choledochorrhaphy) in 146 patients, with a bile leakage rate of 3.8% in T-tube insertion, 8.6% in antegrade stenting and 16.7% in primary choledochorrhaphy, respectively [11]. In our center, LC + LCBDE + intraoperative ENBD + primary duct closure has gained surgeons preference with lower biliary complications, as well as the LTCBDE (Fig. 1). To reduce postoperative complications and improve stone clearance rate, we retrospectively reviewed the experience of 6 years in our single institution with three treatment methods for cholecystolithiasis combined with choledocholithiasis, with emphasis on Tri-scope approach versus LTCBDE and ERCP + LC approach.

**Fig. 1:**
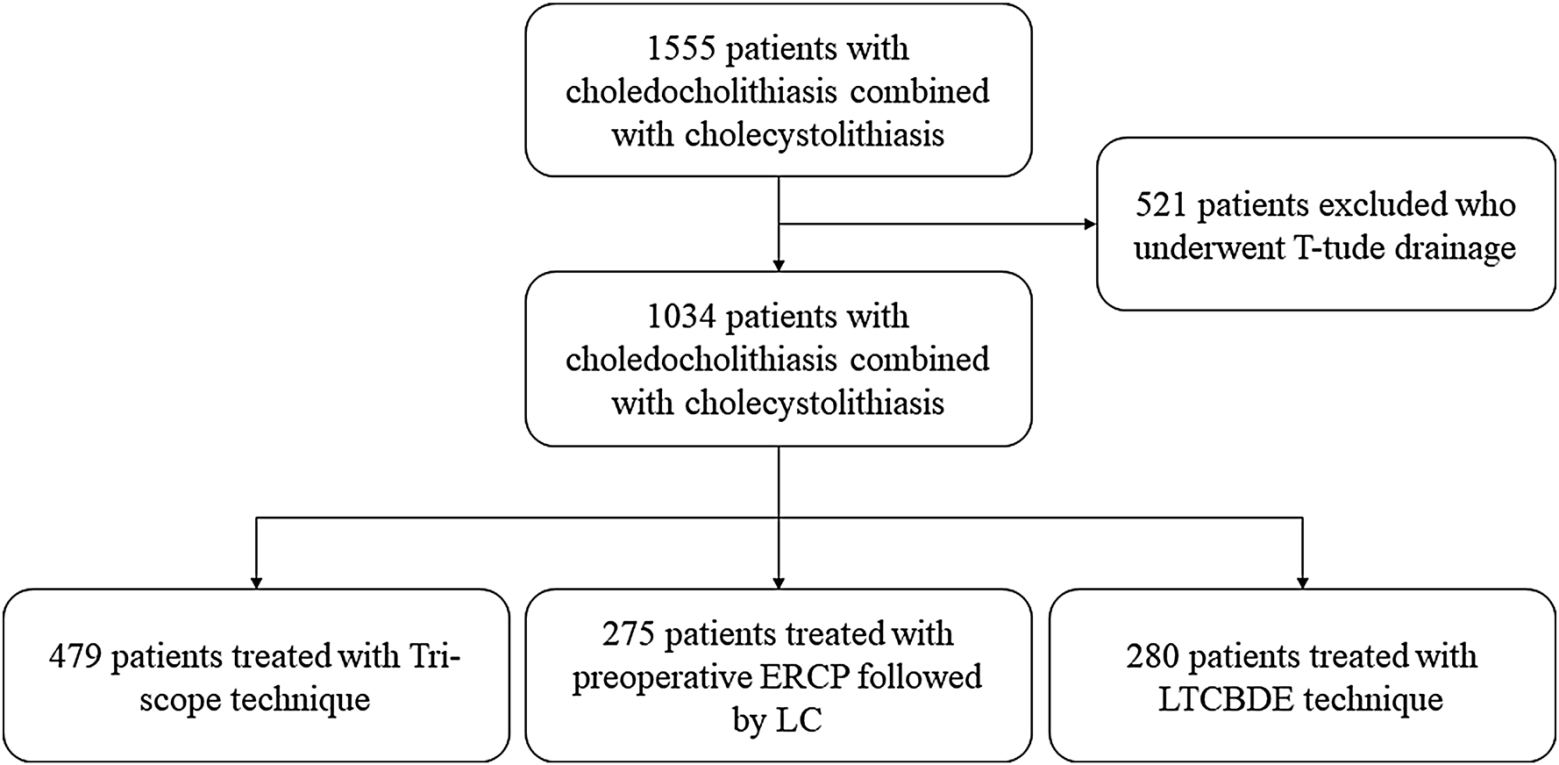
Flow chart of patients with choledocholithiasis combined with cholecystolithiasis

## Methods

### Patients

We performed a retrospective analysis of 1555 consecutive patients who were diagnosed with choledocholithiasis accompanied with cholecystolithiasis from Jan 2013 to Jan 2019. The study was approved by the Ethics Committee of Tongji Hospital, Tongji Medical College, Huazhong University of Science and Technology. The diagnosis of cholecystolithiasis combined with choledocholithiasis was made by clinical presentation, the results of serological tests, hepatobiliary ultrasonography, and/or magnetic resonance cholangiopancreatography (MRCP) findings. Intraoperative cholangiography (IOC) was unavailable in our department due to may increase the risk of contrast medium-induced pancreatitis and equipment problem. Exclusion criteria were: (1) intrahepatic bile duct stones; (2) severe inflammations such as severe acute cholecystitis, acute pancreatitis, acute obstructive suppurative cholangitis and so forth; (3) patients received previous upper abdominal surgery or previous ERCP; (4) biliary tumor. Thus, a total of 1034 patients met the eligibility criteria and were included in this research. Among them, 275 underwent preoperative ERCP + subsequent LC (ERCP + LC group), 759 underwent single-stage treatment including LC + LCBDE + intraoperative ENBD + primary duct closure (Tri-scope group, *n* = 479) and LC + laparoscopic transcystic common bile duct exploration (LTCBDE group, *n* = 280). T-tube was inserted in the setting of compression stone, stenosis of the bile duct and suspected residual stones. The choice of three treatment methods was depend on surgeon’s discretion, preoperative imaging findings and patient’s choice.

All procedures were performed by experienced biliary experts in our center. All data including demographic information, presenting symptoms, preoperative serological data, number and size of stones identified intraoperatively, diameter of CBD and cystic duct, postoperative hospital stay, operating time, time to drain removal, mortality, and morbidity were recorded. The operative time, intraoperative blood loss, operation costs, postoperative hospital stay in ERCP group were determined by summing each of these variates for both ERCP and LC. Additionally, the interval between LC and ERCP varies from 3 to 5 days. The definition of bile leakage was that bile presented in the abdominal drainage or bile was found in ascites by puncture as well as local or general peritonitis was detected. The diameter of CBD and cystic duct was measured by MRCP and intraoperative catheter. The patients were followed up at the outpatient clinic 1 month after the operation and then every 6 months by telephone.

### Surgical strategy

#### ERCP/EST approach

After half an hour of routine intramuscular injection of diazepam and raceanisodamine, the patient was laid in left side or prone position. Then, the ERCP was performed via a duodenal endoscope (Olympus CV-260) in a standard manner by an endoscopist and his first assistant in our own biliary department. Following deep cannulation, retrograde cholangiography, sphincterotomy or balloon expansion (Wil-son-Cook TX-15-A), the CBD stones were extracted by a basket (Wilson-Cook MWB3*6) until no stones were confirmed by repeat cholangiogram. Mechanical lithotripsy was used to retrieve the stones if needed. After complete stone removal, a nasobiliary catheter was routinely placed at the common bile duct. Serum amylase was detected 2 h and 24 h postoperatively and subsequent laparoscopic cholecystectomy (LC) was performed within 3–5 days.

#### Single-stage

After receiving endotracheal general anesthesia, the patient was laid in the supine position. A standard 4-trocar approach was performed for LC, with carbon dioxide pneumoperitoneum at 12 mmHg pressure. Two 10 mm-trocars were placed in umbilicus and below the xiphoid, respectively, another two 5 mm-taocars were introduced in the midclavicular line and right axillary line below the costal margin. The Calot's triangle was carefully dissected and the cystic artery was ligated by absorbable clip, as was the cystic duct. Then, cholecystectomy was performed in a standard antegrade means. Different CBD exploration approaches were as follows:

#### LTCBDE approach

The cystic duct close to gallbladder was clipped and the distal cystic was reserved temporarily so that to temporarily to access common bile duct. The cystic duct was cut transversely at a distance of 1–2 cm to CBD, after which a catheter or balloon was used to dilate the cystic duct. If the diameter of cystic duct < 5 mm and > 3 mm, a 3 mm choledochoscope was inserted through the incision to explore the CBD. If the diameter of cystic duct ≥ 5 mm, we used 5 mm choledochoscope. For patients with the diameter of stone size/cystic duct ≥ 1, we made a T-shaped incision at the confluence of the cystic duct and CBD and used electrohydraulic lithotripsy or biopsy forceps for stone fragmentation. A stone basket and saline irrigation were routinely used to retrieve the stones. After confirming there was no retained stone, the cystic duct was ligated near the CBD by absorbable clip or was sutured.

#### Tri-scope approach

After complete removal of the gallbladder, choledochotomy performed, the length is 1–1.5 cm. Then a 5 mm choledochoscope was inserted into the CBD through the opening to extract the stones until no remnant stones were found in intrahepatic and extrahepatic bile duct. Then a guide wire (MTW-0.027; MTW Endoskopie,Wesel, Germany) was passed through the CBD via choledochoscope, passed the Ampulla of Vater and arrived at the lumen of the descending duodenum, to be later retrieved by biopsy forceps via duodenoscope operated by another experienced endoscopist. Under the guidance of the guide wire, the ENBD tube was slowly and gently sent to 1–2 cm above the choledochal incision. After pulling out the guide wire, the nasobiliary drainage tube was extracted from the body through the nose and was connected to a negative pressure suction ball. The CBD was closed with a 4–0 absorbable suture, after which a silicone drainage tube was routinely positioned beside the Foramen of Winslow.

### Data analysis

We used IBM SPSS 24.0(Inc., Chicago, IL, USA) software to perform statistical analysis. All continuous variables were expressed as mean ± SD and the categorical data were expressed as number and percent (*N* (%)). Continuous data were analyzed by the one-way ANOVA, and categorical data were analyzed by Cochran–Mantel–Haensze-*χ*^2^ test or Fisher’s exact test when appropriate. All analyses were two-sided and *P* < 0.05 was considered to be statistically significant.

## Results

General characteristics, biochemical tests and clinical presentations of these patients are shown in Table 1. There was no difference in terms of demographic characteristics, biochemistry findings, and clinical feature. There was no mortality in three groups. CBD stone clearance rate was 97.5% in ERCP group, 98.7% in Tri-scope group, 99.3% in LTCBDE group. ERCP + LC group had the lowest success rate than Tri-scope group and LTCBDE group. The Tri-scope group had the biggest diameter of stones, amount of stones, and diameter of CBD. LTCBDE group had the least CBD stones and the biggest diameter of CGD. Fourteen cases in ERCP + LC group due to large or compression CBD stones and 4 cases in Tri-scope group on account of stenosis of hepatic bile duct or suspicious remnant stones were converted to T-tube drainage, and stones were cleared completed finally. Meanwhile, 7 cases in LTCBDE group required conversion to Tri-scope approach due to large stones. There were more postoperative complications in ERCP + LC group (3.6%) than LTCBDE group (1.1%) and Tri-scope group (2.2%). Five patients (4 in ERCP + LC group and 1 in Tri-scope group) with pancreatitis recovered with conservative treatment. Three cases appeared with hemorrhage in ERCP + LC group, of which two patients were well-healed with hemostatic and fluid therapy, and one patient was cured with interventional therapy. Totally 8 patients (0.8%) developed bile leakage and recovery after drainage within 1–3 days during hospitalization. Two patients in LTCBDE group and 6 in Tri-scope group required postoperative ERCP due to retained stones. Follow-up time was 6 to 72 months. Four patients in ERCP + LC group and 5 in Tri-scope reported recurrent stones. No patient converted to open surgery in three groups. The results were described in Table 1.

**Table 1:** Demographic and outcomes of patients with cholecystolithiasis combined with choledocholithiasis

	ERCP+LC (*n* = 275)	LTCBDE (*n* = 280)	Tri-scope (*n* = 479)	*p*
Age (year)	51.79 ± 15.68	52.11 ± 15.06	51.65 ± 16.07	0.926
Sex (male/female)	147/128	138/142	268/211	0.457
Preoperative Biochemistry findings				
ALT (U/L)	45.91 ± 28.74	44.83 ± 24.02	42.88 ± 19.20	0.204
AST (U/L)	47.00(22.00,95.00)	41.00 (19.00, 101.00)	50.00(25.00,121.00)	0.082
TBIL (μmol/L)	57.14 ± 79.41	68.08 ± 95.21	61.51 ± 88.84	0.338
DBIL (μmol/L)	13.50(4.75, 34.60)	8.80(3.80,34.45)	8.70(3.95,41.60)	0.627
Albumin (g/L)	39.04 ± 4.80	40.02 ± 6.62	38.51 ± 4.43	0.375
GGT (U)	293.77 ± 287.48	350.60 ± 376.93	319.62 ± 231.12	0.072
ALP (U)	142.00(80.50,202.00)	156.00(107.50,223.50)	145.00(89.00,199.00)	0.032
Leukocyte (10^9^/L)	5.93 ± 2.85	6.09 ± 2.46	6.12 ± 3.18	0.69
Stone characteristics				
Diameter of stones (cm)	0.82 ± 0.46	0.82 ± 0.38	0.93 ± 0.45	< 0.001
Number of stones	1.83 ± 0.94	1.52 ± 0.85	1.99 ± 1.30	< 0.001
Diameter of CBD (cm)	0.81 ± 0.27	0.93 ± 0.28	1.11 ± 0.32	< 0.001
Diameter of CGD (cm)	0.37 ± 0.13	0.50 ± 0.16	0.38 ± 0.16	< 0.001
ASA (%)				0.701
I	61 (22.9)	56 (20.0)	97 (20.3)	
II	182 (68.4)	192 (68.6)	338 (70.6)	
III	23 (8.6)	32 (11.4)	44 (9.2)	
Pain (%)	256 (93.1)	262 (93.6)	459 (95.8)	0.21
Nausea (%)	84 (30.5)	91 (32.5)	150 (31.3)	0.882
Emesis (%)	81 (29.5)	73 (26.1)	111 (23.2)	0.161
Fever (%)	42 (15.3)	43 (15.4)	74 (15.4)	0.998
Jaundice (%)	55 (20.0)	49 (17.5)	86 (18.0)	0.71
Pancreatitis (%)	21 (7.6)	21 (7.5)	38 (7.9)	0.974
Postoperative biochemistry findings				
Leukocyte (10^9^/L)	9.30 (7.53, 11.30)	7.50 (6.17, 8.70)	7.78 (6.92, 10.90)	0.001
TBIL (μmol/L)	19.66 ± 18.24	21.27 ± 16.61	21.20 ± 8.49	0.286
ALT (U/L)	34.81 ± 17.15	35.23 ± 14.31	36.38 ± 20.34	0.467
AST (U/L)	36.69 ± 16.43	37.06 ± 20.49	36.08 ± 12.22	0.697
Hospital stays (days)	14.16 ± 3.88	6.92 ± 1.71	10.74 ± 5.30	< 0.001
Extubation time (days)	3.96 ± 2.21	4.13 ± 1.70	7.23 ± 4.01	< 0.001
Operation time (min)	126.08 ± 42.79	92.31 ± 10.26	99.09 ± 8.46	< 0.001
Postoperative complications				
Pancreatitis (%)	4 (1.5)	0 (0.0)	1 (0.2)	0.024
Bile leak (%)	0 (0.0)	3 (1.1)	5 (1.0)	0.056
Bleeding (%)	3 (1.1)	0 (0.0)	3 (0.6)	0.235
Cholangitis (%)	3 (1.1)	0 (0.0)	3 (0.6)	0.235
Residual stones (%)	7 (2.5)	2 (0.7)	6 (1.3)	0.174
Recurrence (%)	4 (1.5)	0 (0.0)	5 (1.0)	0.156

*ALT* aspartate transaminase, *AST* oxaloacetic transaminase, *TBIL* total bilirubin, *DBIL* direct bilirubin, *GGT* gamma-glutamyl transpeptidase, *ALP* A Lkaline Phosphatase, *CBD* common bile duct, *CGD* cystic gall duct, *ASA* American Society of Anesthesiologists

### ERCP + LC group vs Tri-scope group

There was no statistically significant difference in the diameter of cystic duct. However, the average diameter of CBD in Tri-scope group was significantly larger than ERCP + LC group (1.11 ± 0.32 cm vs 0.81 ± 0.27 cm, *P* < 0.05), so as the diameter of the stones (0.93 ± 0.45 cm vs 0.82 ± 0.46 cm). The intraoperative blood loss in ERCP + LC group was similar in Tri-scope group. The operation duration in Tri-scope group was significantly shorter than ERCP + LC group (99.09 ± 8.46 min vs 126.08 ± 42.79 min, *P* < 0.05), as was the postoperative hospital stay (10.74 ± 5.30 days vs 14.16 ± 3.88 days, *P* < 0.05). The postoperative inflammatory reaction in the Tri-scope was significance lower (7.78 × 10^9^/L vs 9.30 × 10^9^/L, *P* < 0.05).The postoperative complications in ERCP + LC group (3.6%) was more than Tri-scope group (2.2%), but there was no significant difference. Nevertheless, the time of nasobiliary drainage in ERCP + LC group (3.96 ± 2.21 days) was shorter than Tri-scope scope (7.23 ± 4.01 days). There was no difference in postoperative complications. The results were described in Table 2

**Table 2:** The characteristics and outcomes of ERCP + LC group and Tri-scope group

	ERCP + LC (*n* = 275)	Tri-scope (*n* = 479)	*P*
Stone characteristics			
Diameter of stones (cm)	0.82 ± 0.46	0.93 ± 0.45	0.001
Number of stones	1.83 ± 0.94	1.99 ± 1.30	0.077
Diameter of CBD (cm)	0.81 ± 0.27	1.11 ± 0.32	< 0.001
Diameter of CGD (cm)	0.37 ± 0.13	0.38 ± 0.16	0.873
Postoperative biochemistry findings			
Leukocyte (10^9^/L)	9.30 (7.53, 11.30)	7.78 (6.92, 10.90)	< 0.001
TBIL (μmol/L)	19.66 ± 18.24	21.20 ± 8.49	0.117
ALT (U/L)	34.81 ± 17.15	36.38 ± 20.34	0.283
AST (U/L)	36.69 ± 16.43	36.08 ± 12.22	0.56
Hospital stay (days)	14.16 ± 3.88	10.74 ± 5.30	< 0.001
Extubation time (days)	3.96 ± 2.21	7.23 ± 4.01	< 0.001
Operation time (min)	126.08 ± 42.79	99.09 ± 8.46	< 0.001
Intraoperative bleeding (mL)	61.05 ± 62.89	59.13 ± 18.69	0.532
Postoperative complications	10 (3.6)	12 (2.5)	–
Pancreatitis (%)	4 (1.5)	1 (0.2)	0.118
Bile leak (%)	0 (0.0)	5 (1.0)	0.174
Bleeding (%)	3 (1.1)	3 (0.6)	0.791
Cholangitis (%)	3 (1.1)	3 (0.6)	0.791
Residual stones (%)	7 (2.5)	6 (1.3)	0.307
Recurrence (%)	4 (1.5)	5 (1.0)	0.88

### ERCP + LC group vs. LTCBDE group

There was no statistically significant difference the diameter of stones. The diameter of cystic duct in LTCBDE group (0.50 ± 0.16 cm) was larger than ERCP + LC group (0.37 ± 0.13 cm), so as the diameter of common bile duct (0.93 ± 0.28 cm vs 0.81 ± 0.27, *P* < 0.05) and the number of stones in ERCP + LC group were more (1.83 ± 0.94 vs 1.52 ± 0.85, *P* < 0.05). The intraoperative blood loss in ERCP + LC group (61.05 ± 62.89 mL) was significant more than LTCBDE group (42.21 ± 39.89 mL). The operation time in LTCBDE group was significantly shorter than ERCP + LC group (92.31 ± 10.26 min vs 126.08 ± 42.79 min, *P* < 0.05), so as the hospital stay days (14.16 ± 3.88 days vs 6.92 ± 1.71 days, *P* < 0.05). There was no difference in postoperative complications. The results were described in Table 3.

**Table 3:** The characteristics and outcomes of ERCP + LC group and LTCBDE group

	ERCP + LC(n = 275)	LTCBDE(n = 280)	*P*
Stone characteristics			
Diameter of stones (cm)	0.82 ± 0.46	0.82 ± 0.38	0.943
Number of stones	1.83 ± 0.94	1.52 ± 0.85	< 0.001
Diameter of CBD (cm)	0.81 ± 0.27	0.93 ± 0.28	< 0.001
Diameter of CGD (cm)	0.37 ± 0.13	0.50 ± 0.16	< 0.001
Postoperative biochemistry findings			
Leukocyte (10^9^/L)	9.30 (7.53, 11.30)	7.50 (6.17, 8.70)	< 0.001
TBIL (μmol/L)	19.66 ± 18.24	21.27 ± 16.61	0.278
ALT (U/L)	34.81 ± 17.15	35.23 ± 14.31	0.755
AST (U/L)	36.69 ± 16.43	37.06 ± 20.49	0.815
Hospital stay (days)	14.16 ± 3.88	6.92 ± 1.71	< 0.001
Extubation time (days)	3.96 ± 2.21	4.13 ± 1.70	0.324
Operation time (min)	126.08 ± 42.79	92.31 ± 10.26	< 0.001
Intraoperative bleeding (ml)	61.05 ± 62.89	42.21 ± 39.89	< 0.001
Postoperative complications	10 (3.6)	3 (1.1)	–
Pancreatitis (%)	4 (1.5)	0 (0.0)	0.128
Bile leak (%)	0 (0.0)	3 (1.1)	0.257
Bleeding (%)	3 (1.1)	0 (0.0)	0.241
Cholangitis (%)	3 (1.1)	0 (0.0)	0.241
Residual stones (%)	7 (2.5)	2 (0.7)	0.17
Recurrence (%)	4 (1.5)	0 (0.0)	0.128

### LTCBDE group vs Tri-scope group

The diameter of CBD in LTCBDE group was finer than Tri-scope group (0.93 ± 0.28 cm vs 1.11 ± 0.32 cm, *P* < 0.05), but on the contrary to the diameter of CGD(0.50 ± 0.16 cm vs 0.38 ± 0.16 cm, *P* < 0.05).The mount and diameter of stone in Tri-scope were more and bigger than LTCBDE group(0.93 ± 0.45 cm vs 0.82 ± 0.38 cm; 1.99 ± 1.30 vs1.52 ± 0.85, *P* < 0.05). Comparing to LTCBDE group, the hospital stay days and surgery time was longer in Tri-scope group, so as the operation time and intraoperative bleeding (*P* < 0.05). Meanwhile, the drainage time was shorter in LTCBDE group than Tri-scope group (4.13 ± 1.70 days vs 7.23 ± 4.01 days *P* < 0.05). There was no difference in postoperative complications. The results were described in Table 4.

**Table 4:** The characteristics and outcomes of LTCBDE group and Tri-scope group

	LTCBDE (*n* = 280)	Tri-scope (*n* = 479)	*P*
Stone characteristics			
Diameter of stones (cm)	0.82 ± 0.38	0.93 ± 0.45	< 0.001
Number of stones	1.52 ± 0.85	1.99 ± 1.30	< 0.001
Diameter of CBD (cm)	0.93 ± 0.28	1.11 ± 0.32	< 0.001
Diameter of CGD (cm)	0.50 ± 0.16	0.38 ± 0.16	< 0.001
Postoperative biochemistry findings			
Leukocyte (10^9^/L)	7.50 (6.17, 8.70)	7.78 (6.92, 10.90)	0.016
TBIL (μmol/L)	21.27 ± 16.61	21.20 ± 8.49	0.936
ALT (U/L)	35.23 ± 14.31	36.38 ± 20.34	0.406
AST (U/L)	37.06 ± 20.49	36.08 ± 12.22	0.408
Hospital stay (days)	6.92 ± 1.71	10.74 ± 5.30	< 0.001
Extubation time (days)	4.13 ± 1.70	7.23 ± 4.01	< 0.001
Operation time (min)	92.31 ± 10.26	99.09 ± 8.46	< 0.001
Intraoperative bleeding (mL)	42.21 ± 39.89	59.13 ± 18.69	< 0.001
Postoperative complications	3 (1.1)	12 (2.5)	–
Pancreatitis (%)	0 (0.0)	1 (0.2)	1
Bile leak (%)	3 (1.1)	5 (1.9)	0.68
Bleeding (%)	0 (0.0)	3 (0.6)	0.467
Cholangitis (%)	0 (0.0)	3 (0.6)	0.467
Residual stones (%)	2 (0.7)	6 (1.3)	0.74
Recurrence (%)	0 (0.0)	5 (1.0)	0.211

## Discussion

Choledocholithiasis may occur in 10%-18% patients with cholecystolithiasis [1, 12]. The diagnosis of choledocholithiasis is often proved by clinical presentations, liver function testing and imaging examination including MRCP and ERCP [13, 14]. All patients in our center were diagnosed by MRCP and get high accuracy. Intraoperative cholangiography was reported a sensitivity rate of 97% in diagnosing choledocholithiasis [15], but that is not available in our department due to the equipment problem and other reason. In 2016, the EASL(the European Association for the Study of the Liver8) recommended the ERCP + LC as the method for the prevention, diagnosis, and treatment of gallstones [16]. However, ERCP requires two-stage surgery and had higher complications and lower stone clearance [17], as shown the same results in our study. ERCP is a good choice for patients with high risk of anesthesia, having contraindications for MRCP, or with the diameter of CBD less than 8 mm. Short-term complications of ERCP/EST, such as acute pancreatitis, hemorrhage, duodenum perforation and long-term complications such as recurrence of choledocholithiasis, duodenobiliary reflux and papillary stricture due to permanent loss of function of Oddi sphincter have caused of great concern.

With choledochoscope first reported by Leslie [18],single-stage management as a alternative minimally invasive approach to ERCP, plays an crucial role in the treatment of choledocholithiasis. Several randomized clinical trials demonstrated that the single-stage approach had greater advantages in terms of shorter hospital stay and lower cost of hospitalization, which are according with our findings [19–21]. Laparoscopic CBD exploration can be performed either via transcystic approach or through choledochotomy approach, which includes primary duct closure, T-tube drainage, stent drainage, and intraoperative ENBD. The optimal treatment for cholecystolithiasis combined with choledocholithiasis has not yet reached a consensus.

The selection of three methods depends on surgeons’ clinical experience and on an intent-to-treat. One prior report showed biliary related complications occurred in stent drainage much less frequently than that in T-tube drainage and primary duct closure [22].

However, biliary stent drainage has led to new complications of stent displacement and duodenal erosion [23]. Therefore, in our department, we preferred to choose primary duct closure + intraoperative ENBD, with a biliary leakage rate of (1.88%), which is consistent with previous study [24], as an alternative method to stent drainage or primary duct closure. LC + LCBDE + intraoperative ENBD was often performed in patients with a CBD diameter of more than 0.8 cm and without intrahepatic bile duct stones [25]. LC + LCBDE + intraoperative ENBD has shorter hospital stays, lower surgery expenditures, postoperative complications and higher success rate than ERCP + subsequent LC.

LTCBDE has gained wide applicability with the priority of shorter hospital stays, lower hospital costs and fewer postoperative complications over two aforementioned methods [26, 27]. However, the surgical success rate of LC + laparoscopic transcystic CBD exploration varies between 55 and 85% due to cystic duct anatomical features and stone characteristics [28–30]. Based on our study results, LC + LTCBDE was often performed in patients with the diameter of cystic duct larger than 3 mm, the number of CBD stones less than five, or the size of stone smaller than 2 cm, the success rate of which can be up to 93.1% with the availability of 3 mm choledochoscope, electrohydraulic lithotripsy and biopsy forceps.

T-tube drainage has a postoperative complication rate of approximately 15%, which involves T-tube migrations, bile peritonitis after the remove of T-tube, bile leakage, fluid and electrolyte disturbance [31, 32]. If the patient had one of the following characteristics [33].we prefer to choose the T-tube drainage approach: (1) retained of excessive stones or accompanied by intrahepatic duct stones; (2) severe biliary duct infection; (3) combined with bile duct stricture; (4) concomitant biliary pancreatitis; (5) failed ERCP or Tri-scope approach. LC + LCBDE + T-Tube an alternate option if all three other options failed, we can use percutaneous choledochoscope postoperative to remove stone, dilate stenosis and other operations.

In conclusion, three surgical methods of treatment for cholecystolithiasis combined with choledocholithiasis are safe and effective and has its priorities and drawbacks. We should choose the most appropriate surgical methods for each patient according to the preoperative imaging findings.
